# The Effect of Exercise on Immune Response in Population with Increased Risk Factors for Cardiovascular Disease: A Systematic Review

**DOI:** 10.21315/mjms2024.31.5.6

**Published:** 2024-10-08

**Authors:** Nik Siti Nik Zaid, Ayu Suzailiana Muhamad, Mohd Nidzam Jawis, Foong Kiew Ooi, Mahaneem Mohamed, Rohimah Mohamud, Nor Faeiza Hamdan, Normah Jusoh

**Affiliations:** 1 Exercise and Sports Science Programme, School of Health Sciences, Universiti Sains Malaysia, Kelantan, Malaysia; 2 Department of Physiology, School of Medical Sciences, Universiti Sains Malaysia, Kelantan, Malaysia; 3 Department of Immunology, School of Medical Sciences, Universiti Sains Malaysia, Kelantan, Malaysia; 4 Faculty of Sports Science and Coaching, Universiti Pendidikan Sultan Idris, Perak, Malaysia

**Keywords:** exercise, immune response, immunity, immune function, cardiovascular diseases

## Abstract

This systematic review aimed to provide information on existing interventional studies that evaluate the efficacy of exercise in populations with increased cardiovascular disease (CVD) risk factors through immune functional perspectives. A literature search was conducted in four databases: PubMed, Scopus, Taylor & Francis and ScienceDirect from January 2012 to February 2023. The articles were screened and evaluated for quality before data were extracted. The review protocol was registered at PROSPERO (CRD42022321704). In total, 18 studies were included for quality appraisal and synthesised evidence indicated that exercise contributes to enhancing the functioning of both innate and adaptive immune responses, potentially serving as an anti-immunosenescent response to exercise in individuals with elevated CVD risk factors. Furthermore, the review emphasised that exercise, irrespective of its type, intensity or mode, was well tolerated by individuals at increased risk for CVD and may have significant implications in generating anti-inflammatory effects.

## Introduction

Exercise has become progressively critical in the healthcare division to combat and prevent epidemic diseases, including cardiovascular disease (CVD). CVD is associated with a large mortality rate (1). It is estimated that CVD will remain a leading cause of early mortality by 2030, affecting nearly 23.3 million individuals around the world (2). Increased risk of CVD has been linked to poor health status, dysregulated and weakened immune system (3). The immune system involves the precise coordination of various cell types and molecular messengers through complex and redundant mechanisms of action. This underlying complexity of the immune system is essential in defending the body against pathogenic microorganisms such as bacteria, fungi, viruses and protozoa (4). The immune system components are comprised of cellular and soluble elements summarised in Table 1 (5). The attempts of infectious foreign agents to invade the body instantly trigger the innate system which is the first line of defence. The failure of the innate system results in infection, which further stimulates the adaptive system to aid in recovery. The adaptive system responds with the proliferation of cells that either attack the invader or produce specific defensive proteins that aid in combating the pathogen through various mechanisms of action (6).

The immune system is very responsive to exercise-generated stimuli, specifically the intensity and duration of exercise that reflects the extent of physiological stress. The benefits of exercise may result from the direct effect on immune response modulations or through its physiological effects (3). Studies have shown that exercise is a powerful lifestyle intervention that is used in earnest to improve immunity and other health outcomes in various populations such as obese (7), hypertension (8) and diabetic (7) individuals. For instance, regular moderate-intensity exercise is associated with enhanced immune function and decreased risk of infection as exercise induces physiological changes in the immune system (5). On the other hand, prolonged bouts of strenuous exercise result in transient depression of white blood cell (WBC) functions.

To date, there is limited evidence regarding the effect of exercise on immune response in populations with increased risk of CVD risk factors and there is compelling but mixed data on its response in the various CVD populations. Regarding the inconsistent evidence and the increasing interest in the role of exercise in CVD management, the key purpose of this systematic review is to synthesise the currently available evidence and clarify the conflicting evidence between studies. To the best of our knowledge, no previous reviews have been conducted on the effect of exercise on immune parameters in populations with increased risk of CVD. Therefore, to address these issues, this systematic review focuses on existing interventional studies to evaluate the efficacy of exercise in populations with increased CVD risk factors from immune functional perspectives.

## Method

### Study Design

The search was conducted according to the Preferred Reporting Items for Systematic Reviews and Meta-Analyses (PRISMA) guidelines (9). The review protocol was registered at PROSPERO (CRD42022321704) on 1 May 2022 and can be assessed at PROSPERO (https://www.crd.york.ac.uk/prospero/).

### Search Strategy

Related studies were searched electronically using the following accessible databases: PubMed (https://pubmed.ncbi.nlm.nih.gov/), Scopus (http://www.scopus.com), Taylor & Francis (https://www.tandfonline.com/) and ScienceDirect (https://www.sciencedirect.com/) from January 2012 to February 2023 in which the following keywords were used during the search: ‘exercise AND immune response’.

### Inclusion and Exclusion Criteria

The publications were screened for eligibility stepwise based on the study objective. The inclusion and exclusion criteria were based on population, intervention, control/comparator and outcome (PICO), summarised in Table 2. The inclusion criteria were: i) any studies employing exercise intervention like aerobic, anaerobic, resistance, etc.; ii) any original articles including immune system parameters as the outcome measure; iii) any articles involving adults with increased risk of CVD and iv) only randomised controlled trials and experimental studies on humans.

Articles with the following criteria were excluded: i) non-peer-reviewed articles; ii) any articles involving non-human participant testing; (iii) any articles published in languages other than English; (iv) any studies combining exercise with supplementation or other therapies; (v) studies with no control/other exercise mode group; (vi) studies with no full-text access and (vii) conference abstracts/proceedings, study protocols, letters, opinion articles and duplicated reports.

### Study Selection

After searching the above-mentioned databases, the results were exported to the reference manager software Mendeley Desktop (version 1.19.8, Mendeley, London, United Kingdom) to remove duplications using the built-in function ‘check for duplicates’. The number of duplicates was recorded according to the search output. In addition, no cross-referencing was performed on related previously published studies.

Next, the titles and abstracts of retrieved articles were reviewed using the criteria to determine whether full texts were required for further analysis. Abstracts that fulfilled the inclusion criteria were retrieved for further screening before attaining full-text articles. Each full-text manuscript was evaluated systematically according to the: i) objective/s of the study; ii) characteristics of the study (study design, participants, age and sample size); iii) information about intervention (type of intervention, length of intervention, follow-up period or mode of exercise); iv) targeted outcome/s (mean and SD of immune responses at baseline, post-intervention and/or changes in immune responses from baseline to the end-of-trial) and v) main findings. In the final step, the full text of the selected studies was carefully reviewed to determine the final eligible publications. The outcomes extracted from these studies were not combined, reanalysed or changed due to the nature of this systematic review. Two independent investigators (NZNS and NFH) performed all these procedures and any disagreements to include certain articles were resolved by discussion with a third reviewer (ASM, MNJ, FKO, MM, RM, NJ). Discussions were conducted among the authors before reaching any consensus.

### Data Extraction

The outcome variables were compiled using Microsoft Excel. For each of the included studies, parameters such as the first authors’ surname and study year, sample size, study population, type of exercise, comparable group, intervention duration/sampling time and major findings were noted. No attempts were made to contact the authors for additional information. If a study provided multiple data at different time points, only the latest was considered.

### Quality Assessment

The methodological quality of each paper was evaluated by the authors (NFH, NZNS and ASM) using Quality Appraisal according to (10) based on the rating scale between group comparisons, point estimated and variability biases in the studies. The rating scales were study design (C1): RCT = 1, otherwise = 0; sample size (C2): large = 1, small = 0; selection of samples (C3): random selection or lack of bias = 1, non-random or convenience sample or presence of bias = 0; analyses (C4): robust (such as multivariate analyses) = 1, otherwise = 0. Each item was given points and the scores were determined when the criteria were internally valid and had sufficient statistical information. A score of more than or equal to 2 (moderate to high) was considered to represent a high-quality study. A low-quality study was represented with a score of 1 and 0. Differences in opinion on any quality appraisal item score were resolved through discussion until a consensus was reached.

## Results

### Search Results

**Figure 1:**
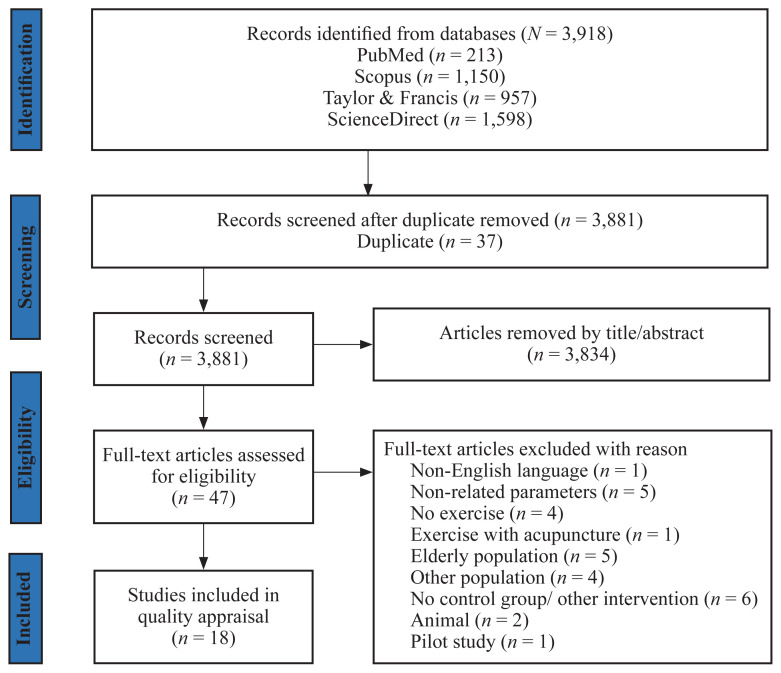
PRISMA flow diagram for the study selection

A PRISMA flow diagram showing the stages of articles’ screening and selection is shown in Figure 1.

The initial search from the database identified 3,918 articles. First, 3,834 articles were excluded based on the screening of titles and abstracts. Next, 37 duplicated articles were excluded. After these exclusions, the full text of 47 potentially relevant articles was screened, leading to the subsequent removal of 29 articles. Finally, a total of 18 studies were deemed eligible for quality appraisal and data extraction. Pertinent data extracted from each article are presented in Table 3.

### Study Characteristics

The 18 studies included were conducted between 2015 and 2023, and comprised 613 participants, summarised in Figure 1.

### Participant Characteristics

The studies were exclusively comprised of adults. Six studies included only males (11–16), six studies included only females (17–22), and the remaining six studies involved both males and females (23–28).

There was a total sample of 546 participants from all articles. The articles consist of various populations with increased risk of CVD: sedentary (*n* = 5) (14, 15, 18, 24, 25), overweight-obese (*n* = 10) (12, 13, 16, 17, 19–22, 26, 27), sedentary-obese (*n* = 2) (11, 23) and pre-hypertension (*n* = 1) (28).

### Intervention Characteristics

From the 18 articles taken into account, five studies implemented moderate-intensity exercise (15, 18, 22, 27, 28), three implemented high-intensity exercise (13, 14, 16), four studies implemented both moderate and high-intensity exercise (11, 12, 23, 24) and one study implemented progressive loading moderate to high-intensity exercise (26). combined aerobic and resistance training was carried out in four studies (17, 19–21).

All studies included immune parameters and were summarised in Table 3. Six studies involved WBC or leukocytes (12, 15, 18, 19, 23, 24), nine studies involved monocytes (12, 14, 15, 17, 19, 23–26). Lymphocytes and neutrophils were involved in six (12, 14, 15, 18, 19, 24) and four (18, 19, 23, 24) studies, respectively. One study measured the thymus and spleen index (27). IL-6 was involved in six studies (11, 12, 16, 23, 24, 26), four studies involved IL-10 (13, 19, 22, 23), seven studies involved TNF-α (13, 17, 20, 22–24, 26), one study involved IFN-γ (11). Two studies each were related to T cells (23, 28), NK cells (14, 21), NK cells and IgA (17, 21) and IgM (17, 21), respectively.

### Study Quality

The quality of the studies was assessed individually and presented in Table 4. Based on the quality appraisal, only three studies were scored as low-quality, mainly due to study design and no randomisation (14, 20, 26).

### Effects of Exercise on Immune Parameters

Table 5 summarises the effects of exercise on selected immune parameters which were investigated in all studies, i.e. WBC, monocyte, lymphocytes, T cells, neutrophils, NK cells, IL-6, IL-10, TNFα, IFN-γ, IgA, IgG, IgM, thymus and spleen index.

### Exercise-Induced Effects on Cytokines (IL-6, IL-10, TNF-**α**)

A total of six studies investigated the effect of IL-6 in exercise groups. Only three studies showed significant improvement in IL-6 levels in high-intensity interval exercise (HIIE) and exhaustive exercise (ES) at: i) post-intervention (12), ii) immediately post and iii) 1 h post-low-volume HIIE and moderate-intensity continuous exercise (MICE) (11): (i) immediately after exercise in HIIE and ES group (13), (ii)1 h after high-intensity interval training (HIIT) and (iii) 24 h after HIIT training. In contrast, one study reported a significant reduction of IL-6 after 1 h post-exercise in the active group that performed aerobic exercise (26). Meanwhile, two other studies reported no significant changes in IL-6 levels after 2 weeks of HIIT and moderate-intensity interval training (MICT) (23) and after 10 weeks of HIIT and MICT (24).

One study showed that there was significant improvement in IL-10 levels immediately after exercise in the HIIE group (13) and after 12 weeks of combined aerobic and resistance exercise (19). However, there were two other studies that reported that there were no significant changes in plasma IL-10 levels after five menstrual cycles in low and high-dose aerobic exercise (22) and also after 2 weeks of HIIT and MICT (23).

According to one of the studies, it was reported that there was a statistically significant improvement in TNFα from the baseline in the ES group at post-exercise (12). Similarly, other studies reported a significant improvement in TNFα after five menstrual cycles in the high-dose exercise group (22) and immediately after exercise in the exhaustive exercise group (13). In contrast to these findings, one study reported a significant reduction of TNFα in the exercise groups with high and low-dose aerobic exercise (22) after 10 weeks of combined CHIIT (20) and after 12 weeks of combining aerobic and resistance exercise (17). A study has also reported that there were no significant changes in TNFα after 2 weeks of HIIT and MICT (23), 1 h after aerobic exercise (26) and after 10 weeks of HIIT and MICT (24).

Upon monitoring the IFN-γ level in one of the studies, it was observed that there was a statistically significant decrement in IFN-γ level immediately after exercise in the HIIE group and remained lower level 1 h post-HIIE (11). However, the MICE group reported a significant increase in IFN-γ levels from the baseline immediately after and 1 h post-MICE.

Exercise-induced Effects on Immunoglobulin

It was reported from one study that there was no significant difference in IgA at 12 weeks post-intervention, while IgG and IgM concentrations were significantly reduced in both aerobic and resistance groups (21). These findings did not align with the results of another study, which reported a significant improvement in IgA concentration in an exercise group that performed 12 weeks of combined aerobic exercise and resistance training (17). The authors also reported no significant changes in IgM in the exercise group (17).

### Exercise-Induced Effects on Thymus and Spleen Index

Only one study reported a significant increment in thymus and spleen index in aerobic exercise among 80 obese individuals (27).

### Exercise-Induced Effects on Leukocytes (Monocytes, Neutrophils)

Two studies reported a statistically significant improvement in WBC immediately after and 1 h after exercise in all exercise groups (15). WBC also improved immediately after exercise and 30 min after exercise in the moderate-intensity interval exercise (MIIE) and HIIE groups (12). But, in another study, no statistically significant changes were observed in WBC after 2 weeks of HIIT and MICT (23). Some other studies have also reported a statistically significant reduction in WBC in the aerobic dance group after 8 weeks of intervention (18) and after 12 weeks of combined aerobic and resistance training (19).

The studies about monocytes also had varied findings. According to two of them, statistically significant improvement in monocytes was observed immediately after exercise in all groups that measured the acute response (12, 15). Another study showed significant improvement in monocytes 2 min–5 min post-exercise in the maximal intensity cycling group (14). In addition, one study found significant improvement in monocyte phagocytosis in both HIIT and MICT groups after 10 weeks of intervention (24). Meanwhile, there were no significant change scores in CD14^+^ monocytes in all groups after 2 weeks of intervention in this study (23, 26, 28). No significant change score in monocytes in all groups was seen after 2 weeks of intervention in the study carried out by Mazur et al. (28).

Regarding neutrophils, two studies showed no significant differences in neutrophils after 2 weeks of HIIT and MICT (23) and after 10 weeks of HIIT and MICT (24). Two studies found a significant reduction in neutrophils after 12 weeks of combined aerobic and resistance training (19) and after 8 weeks of aerobic dance exercise (18).

### Exercise-Induced Effects on Lymphocyte Subsets

One study stated that there was a statistically significant improvement in lymphocyte counts immediately after exercise and 1 h after exercise in all groups (15), 2 min–5 min post-exercise in maximal intensity cycling (14), after 12 weeks of combined aerobic and resistance training (19), and immediately post-exercise (12). In contrast, it was found from two studies no statistically significant difference was seen in lymphocytes after 8 weeks of aerobic dance (18) and after 10 weeks of HIIT and MICT (24).

Two studies concluded that there were no significant differences in T cells after 2 weeks of HIIT and MICT (23) and after 12 weeks of aerobic training (28). In a study about NK cells, two studies showed statistically significant improvement in NK cell activity after 12 weeks of aerobic exercise and resistance training (21) and 2 min–5 min post-exercise in maximal intensity exercise (14).

## Discussion

This systematic review summarised the available literature on the effect of exercise on immune parameters in populations with increased risk of CVD. The primary findings showed that exercise, regardless of its types and modes, may improve the function of the innate and adaptive immune response as a potential anti-immunosenescent response to exercise in populations with increased CVD risk factors. Table 6 summarises the immune actions of selected parameters.

### Exercise-Induced Effects on Cytokines (IL-6, IL-10, TNF-**α**, IFN-**γ**)

Interleukin-6 (IL-6) is a cytokine responsible for inflammation and plays a vital role in the immune response to stress, including the onset of infection. It is also involved in the generation of stimuli due to exercise (29). It plays an important role in exercise physiology by regulating energy metabolism, inflammation, hormone release, and immune system function (30). A previous study conducted in eight obese men reported a significant increment (*P* < 0.05) of the IL-6 level from pre- to post-test in the HIIE group (from 8.5 ± 2.8 pg/mL to 10.9 ± 1.1 pg/mL) as well as in the ES group (from 8.3 ± 1.7 pg/mL to 11.2 ± 0.8 pg/mL) (13). Similarly, in a randomised crossover trial conducted by de Souza et al. (11), it was observed that there was a significant improvement in IL-6 level immediately post and 1 h post-low-volume HIIE and MICE in 10 sedentary obese males and in HIIE and ES of overweight-obese males post-intervention (12).

This is in contrast to the study conducted by Douglas et al. (26), who reported a significant reduction of IL-6 levels after 1 h post-exercise in overweight to obese adults in the active group who performed moderate to vigorous intensity aerobic exercise. HIIT has been shown to reduce IL-6 levels in overweight men, possibly due to improved insulin sensitivity and decreased adipose tissue inflammation (31). Additionally, weight loss resulting from HIIT may contribute to reducing the levels of IL-6 (32). Also, it has been observed that HIIT increases lipolysis and fatty acid oxidation in adipose tissue, which may further contribute to the increase in IL-6 levels (33). Elevated plasma concentration of IL-6 subsequently stimulates the differentiation of B-cells, inflammation, and acute-phase response to promote a range of benefits in vascular reactivity and the suppression of pro-inflammatory cytokines. This may reduce the incidence of disease (34). IL-6 is known to increase the number of immune cells in the blood, including neutrophils and lymphocytes, which can help to protect the body against infection during and after exercise (30).

The study which investigated the chronic effect of IL-6 in 33 inactive adults with obesity and 27 inactive adults, respectively, reported no significant changes in IL-6 levels for both HIIT and MICT groups after 2 weeks and 10 weeks (*P* < 0.05) (23, 24). There could be several reasons why IL-6 levels did not change. One possibility is the small sample size, in which detecting significant changes could not be recorded (35). Another possibility is individual variations in response to exercise and differences in baseline IL-6 levels. This could also contribute to the lack of significant change (36).

These conflicting findings in the same obese population may be attributed to variations in study methods, especially the type of exercise stimulus. Exercise duration and intensity are cited as principal factors in stimulating IL-6 synthesis and secretion by skeletal muscle (34). Therefore, one out of three acute studies that employed high-intensity and exhaustive exercise protocols lasting approximately 1 h in duration reported significant improvement in IL-6 (11–13).

In addition, physical fitness influences plasma IL-6 concentration because it was seen that prolonged exercise in untrained individuals led to a reduction or depletion in intramuscular glycogen (34). de Souza et al. (11) and Dorneles et al. (13) confirmed this by reporting increased IL-6 in the sedentary and obese men population following a single bout of HIIE, MICT and exhaustive exercise session. In obese individuals, this response may be amplified due to chronic low-grade inflammation in adipose tissue, which can lead to elevated baseline levels of IL-6 (37). Although the exact mechanism remains unclear, an increase in circulating IL-6 following exercise is a crucial physiological response. IL-6 is linked to many biological functions.

Interleukin-10 (IL-10) is an anti-inflammatory cytokine that intermediates the communication between immune and nonimmune cells as an immune mediator to inhibit the exacerbation of the pro-inflammatory response (38). IL-10 was measured in three of the reviewed studies. In 2016, Dorneles et al. (12) conducted a crossover experimental study and reported a significant two-fold improvement in IL-10 levels immediately after exercise in 8 obese men for HIIE and ES compared to before exercise (*P* < 0.05). It has been observed that exercise increases IL-10 levels in the body, which may contribute to the anti-inflammatory effects of exercise (39–41). The increase of IL-10 during exercise is likely mediated by a complex interplay between various signalling pathways and immune cells. Exercise can activate immune cells through increased metabolic demands like changes in blood flow and mechanical stress (41), the release of exercise-induced hormones like cortisol (5) and the adenosine pathway (42).

However, Haley et al. (22) evaluated the efficacy of home-based treadmill aerobic exercise in 116 premenopausal women at high risk for breast cancer and showed no significant improvement (*P* < 0.05) in IL-10 levels in all groups after five menstrual cycles. Similarly, there was no significant improvement in IL-10 levels in 33 inactive adults with obesity after 2 weeks of HIIT and MICT in a randomised control trial conducted by Barry et al. (23). These studies did not report any effect of exercise on IL-10 serum either (43, 44).

This disagreement is speculated due to the differences in interventional duration and exercise protocols employed in each study. Unsurprisingly, there was no counter-effect of IL-10 levels as the protocol did not consider the muscle-derived IL-6 release either. Cabral-Santos et al. (45) reported that the exercise duration is the single most important factor determining the magnitude of exercise-induced increase of plasma IL-10. Therefore, it is likely that the interventional duration is one of the limiting factors for not inducing an anti-inflammatory response in the obese cohort (46). A longer training period would allow further physiological adaptation that might show more robust effects. It is important to note that cytokine responses to exercise are complex and may involve multiple cytokines and immune cells.

TNF-alpha (TNFα) is a pro-inflammatory cytokine. Its function depends on the context and concentration of the cytokine. At low concentrations, TNFα can promote cell survival, tissue repair and immune cell activation, whereas at high concentrations, TNFα can induce cell death, tissue damage and inflammation (47). Three studies reported a significant reduction of TNFα in exercise groups (17, 20, 22). Moderate and high-intensity exercise decreases the levels of systemic pro-inflammatory cytokines, including TNF-alpha (TNFα) (48–50). In contrast, another three studies reported no significant changes in TNFα (23, 24, 26). The mechanisms underlying this positive effect are thought to be multifactorial and may involve several processes. The release of anti-inflammatory cytokines, such as IL-10 and transforming growth factor-beta (TGF-β) during exercise suppresses the production of TNFα (51). Exercise can improve the function and activity of the sympathetic nervous system (SNS) and the hypothalamic-pituitary-adrenal (HPA) axis. The HPA modulates the production and release of TNFα and enhances the resolution of inflammation (52). Exercise also induces the production of heat shock proteins (HSPs), which are stress-inducible proteins that can suppress the production of TNFα and enhance the activity of immune cells, such as NK cells and T cells (53).

While exercise decreases the levels of TNFα, three studies reported a statistically significant improvement in TNFα from the baseline (12, 22), which may reflect the activation of the immune response to tissue damage and stress. The mechanisms underlying this effect are not fully understood but may involve several feasible mechanisms. Exercise can lead to muscle damage and the release of damage-associated molecular patterns (DAMPs). These activate immune cells, such as macrophages, to produce pro-inflammatory cytokines, including TNFα that initiate the repair and regeneration of damaged tissue and activation of immune cells at the site of injury (54). Exercise can induce the production of reactive oxygen species (ROS) (55) and the expression and activity of Toll-like receptors (TLRs) (56). These activate immune cells and induce the production of TNFα. However, the increase in TNFα after exercise is usually transient and returns to baseline levels or decreases below baseline levels after a few hours (57).

IFN-γ is produced by immune cells in response to infection or inflammation (58). One study demonstrated a statistically significant decrement in IFN-γ levels from the baseline immediately after exercise in the HIIE group and remained low 1 h post-HIIE (11). This finding is in line with previous studies (59, 60). However, the MICE group reported a significant increase in IFN-γ levels from the baseline immediately after and 1 h post-MICE. Different findings in high and moderate-intensity groups were supported by previous studies (11). The increase in IFN-γ levels after exercise is thought to enhance the immune response by activating surface receptors of immune cells such as T cells, B cells and natural killer (NK) cells (61) and increasing their expression. This promotes inflammation, which can assist in recruiting immune cells to the site of infection or inflammation (62). Antigen presentation by cells like dendritic cells is enhanced, promoting T cell activation (63). It also promotes antibody production by B cells, which can help to counteract pathogens and prevent their infection (63). This mechanism of action aids in improving the body’s ability to fight off infections, reduce the risk of developing certain diseases and may also provide some health benefits associated with regular physical activity.

In contrast, the decrease in IFN-γ levels after exercise may be due to several factors. One possible explanation is that exercise can cause a shift in the balance of immune cell types, such as a decline in IFN-γ-producing T cells and an increase in other types of immune cells (5). Furthermore, exercise-induced stress can lead to the production of anti-inflammatory cytokines, which may also contribute to the decrease in IFN-γ levels (41).

### Exercise-Induced Effects on Immunoglobulin

IgA, or immunoglobulin A, is an antibody that is responsible for the immune response at mucosal surfaces. Yoon et al. (21) conducted an experimental study to investigate the effects of aerobic and resistance exercise in obese postmenopausal women. Thirty participants were randomly assigned to either aerobic exercise, resistance exercise or control group for 12 weeks. In all groups, no noteworthy difference in IgA concentration was found. these findings were not in line with the studies analysed in systematic review and meta-analysis (64). Similarly, Park et al. (17) stated that there was a significant enhancement in IgA concentration in the exercise group that performed a combined aerobic exercise and resistance training for 12 weeks in postmenopausal middle-aged women with abdominal obesity. Exercise duration may also influence the effect on IgA levels. Some studies suggest that longer durations of exercise may be more effective at increasing IgA levels (65).

Research has also shown that regular exercise can help in the improvement of the body’s ability to defend against infections at mucosal surfaces, which may help to reduce the risk of respiratory and gastrointestinal infections (30). For example, it is seen that exercise increases the binding affinity of IgA antibodies to pathogens, which can help to improve their ability to neutralise and eliminate pathogens (66). The release of certain hormones, such as cortisol and epinephrine, during exercise increases the levels of IgA in saliva and mucosal secretions (67). In addition, exercise can also increase blood flow to the mucosal tissues, which may help to enhance the transport of IgA and other immune cells to these tissues (66).

IgG (immunoglobulin G) is the most abundant antibody in the blood, and IgM (immunoglobulin M) is the first antibody produced by the body in response to an infection or antigen. Yoon et al. (21) also reported a significant reduction in IgG and IgM concentration in both aerobic and resistance groups. Chronic intense exercise or overtraining may lead to an impaired immune response by decreasing IgG and IgM concentration. In contrast, Park et al. (17) reported no significant changes in IgM concentration in all groups. These findings conclude that postmenopausal women with obesity and abdominal adiposity experienced inconsistent findings of IgA, IgM and IgG (17, 21). Nevertheless, the effect of exercise on IgM, IgG and IgA concentrations can depend on various factors such as the individual’s fitness level, body fats, and overall health status. IgG and IgM increased by exercise but decreased following 12 weeks of aerobic and resistance exercise (21). This suggests that immunoglobulin inhibition is affected by the dysregulation of the hormone in the menopausal state due to the increase in the male hormone, testosterone increases, and estrogen decreases. This changes the fat distribution in the female body (68).

### Exercise-Induced Effects on Thymus and Spleen Index

The thymus and spleen are organs of the immune system that play crucial roles in immune function. The strength of immune function can be approximately estimated through the extent of the thymus and spleen index depending on the level where the lymphocytes proliferate (69). Li et al. (27) assessed the effect of medium-intensity aerobic exercise in 80 obese patients and reported a significant improvement in cellular immune function of the thymus and spleen index. Some studies have suggested that moderate-intensity exercise can increase the thymus index, which is a measure of the thymus gland’s size or weight, as a result of increased production of thymic hormones and enhanced thymocyte differentiation (70). In contrast, some studies have suggested that acute, high-intensity exercise can cause a transient decrease in the spleen index, which is a measure of the spleen’s size or weight, due to splenic constriction or temporary redistribution of immune cells to the circulation (71). This suggests that aerobic exercise like brisk walking and jogging can effectively improve the immune system in obese volunteers. However, human studies addressing the cellular immune function, such as the thymus and spleen index’s response to exercise, are scarce and warrant more research to understand the mechanisms. The effect of exercise on the thymus index can also depend on age, with older individuals experiencing less increase in thymus size in response to exercise (72).

### Exercise-Induced Effects Leukocytes (Monocytes, Neutrophils)

Leukocytes, also known as white blood cells, are produced in the bone marrow and circulate in the blood and lymphatic system. They detect and destroy pathogens, infected cells and abnormal cells (6). Two studies reported a statistically significant improvement in leukocytes in all exercise groups that measured acute effect (15, 16). These findings aligned with the previous studies (73–75). It is normal for leukocyte levels to increase after exercise, especially during intense or prolonged physical activity. The degree and duration of increase in leukocytes can vary depending on the type, intensity, and duration of exercise, as well as the individual’s fitness level and overall health. This temporary increase is a part of the body’s natural response to stress to boost the immune system and prevent infection (5). In most cases, leukocyte levels will return to normal within a few hours after exercise. However, if the increase is significant or persists for an extended period, it may indicate an underlying health issue (5).

However, one study reported no statistically significant changes in leukocytes post-intervention in HIIT and MICT groups (23), while two studies showed a statistically significant reduction in leukocytes in the aerobic dance group (18) and in the aerobic and resistance exercise group (19). It is possible for leukocyte levels to not change significantly after HIIT exercise in some individuals. In some cases, the magnitude of the exercise-induced leukocyte response may not be large enough to be detectable by routine blood tests, or other compensatory mechanisms within the body may counteract the initial response (76).

Exercise can cause a temporary decline in the number of circulating leukocytes. During exercise, it is caused by the blood redistribution from the spleen and other organs to the muscles that are working (77), hormonal changes of cortisol that suppress leukocyte production (5) and temporary removal of leukocytes from circulation as they become more adherent to the walls of blood vessels, and increased tissue migration (78). This mechanism of action suggested that the temporary decrease in leukocyte levels after exercise may be due in part to the migration of leukocytes from the bloodstream into tissues, where they can help repair any damage caused by exercise-induced inflammation (76). Additionally, some studies have suggested that the decline in leukocyte levels may be due to the suppression of immune cell production and function caused by high levels of pro-inflammatory cytokines (79).

Monocytes are a type of white blood cell that plays an important role in the immune system by helping to combat infection and inflammation. We found that HIIT workouts had no significant change in monocytes in all groups in the studies (23, 26, 28). In contrast, two studies reported improvement in monocytes immediately after exercise in all groups that measured the acute response (12, 15), while Gustafson et al. (14) reported notable improvement in monocytes 2 min–5 min post-exercise in the maximal intensity cycling group. Similarly, one study reported significant improvement in monocyte phagocytosis in both HIIT and MICT groups after 10 weeks of intervention (24). This finding was similar to another study that also stated that mean monocyte count increased immediately after the acute HIIT, returned to resting levels 3 h post-exercise, and completely returned to resting levels 6 h post-exercise (80). Monocyte counts generally increased after HIIT as a part of the immune response to exercise-induced stress (81). The exact mechanisms underlying this response have not been completely deciphered, but it is thought that the stress of HIIT can lead to the release of certain hormones, such as cortisol. These hormones can stimulate the production and release of monocytes from the bone marrow into the bloodstream. Additionally, the mechanical stress of exercise and the resultant tissue damage can also trigger the immune system to mobilise monocytes to the site of damage to aid in tissue repair and recovery.

Neutrophils are the most abundant type of leukocytes and are capable of releasing enzymes and chemicals that help to destroy pathogens through phagocytosis (82). Two studies reported no noteworthy differences in neutrophils after chronic HIIT and MICT (23, 24). The kinetics of the neutrophil’s response are influenced by the intensity and duration of exercise, where it is highly responsive to acute exercise and accounts for the majority of exercise-induced leukocytosis (83). High-intensity exercise may cause the neutrophil count to double in the order of minutes. Prolonged bouts of endurance exercise (55% VO2max, 164 min) may cause neutrophil numbers to increase three to fourfold after exercise, reaching peak values immediately after exercise before slowly declining to baseline levels 24 h later (84).

### Exercise-Induced Effects on Lymphocyte Subsets (T Cells and Natural Killer Cells)

Lymphocytes are produced in the bone marrow and mature in the thymus gland, lymph nodes and spleen. They provide long-term protection against future infections by recognising and responding to previously encountered pathogens. Current studies found that HIIT intervention led to statistically significant improvement in lymphocytes immediately after exercise and 1 h after exercise in all groups (15). Similarly, another study deduced that there is a significant increase in the number of lymphocytes 2 min–5 min post-exercise in the maximal group (14) and also reported remarkable improvement in lymphocytes post-exercise in overweight and obese (12). In contrast, two studies deduced that there was no statistically significant difference in lymphocytes in all groups (18, 24). Exercise can activate lymphocytes through several mechanisms, including the release of stress hormones such as adrenaline and cortisol, changes in pH and temperature and mechanical stress on the cells. During exercise, lymphocytes are mobilised from the lymphatic system and enter the bloodstream. This results in a temporary increase in the number of circulating lymphocytes. The increase is believed to be a part of the body’s immune response towards the stress of exercise due to the release of certain hormones, such as adrenaline and cortisol (85).

T cells are lymphocytes, small in size, that are involved in cell-mediated immunity. Two studies reported no significant differences in T cells after 2 weeks of HIIT and MICT (23) and 12 weeks of aerobic training (28). This finding contradicts the reports from previous studies. Several human and animal studies have shown that HIIT can increase the number of T cells in the bloodstream and improve their activity (86–88). The specific mechanisms by which HIIT affects T cells are not yet fully understood. Still, it is believed that the physical stress of high-intensity exercise may stimulate the production and release of T cells from the thymus gland and lymphoid tissues (86). In addition, the release of certain hormones, such as adrenaline and cortisol, during exercise may also affect the impact of HIIT on T cells (89).

NK cell is also known as large granular lymphocytes that play a major role in defending the host from both tumours and virus-infected cells. The present study showed a statistically significant improvement in NK cell activity in all groups after 12 weeks (21). Similarly, another study showed a statistically significant improvement in NK cell count 2 min–5 min post-exercise in the maximal intensity cycling group (14). Acute bouts of exercise are believed to increase the number and activity of circulating NK cells, which can enhance their ability to fight infections and tumours. This effect is thought to be mediated by several mechanisms, including the release of stress hormones, such as epinephrine and norepinephrine, that enhance NK cell cytotoxic activity against target cells (90) and the production of cytokines and chemokines, such as IL-6. Cytokines and chemokines promote NK cells’ proliferation and activation and their receptors’ expression. These receptors are responsible for the recognition and killing of aberrant cells, which are involved in the recognition and killing of abnormal cells (91). Exercise can also enhance the trafficking and redistribution of NK cells from peripheral tissues to circulation, which can increase their accessibility and availability to target and destroy abnormal cells (92).

### Consideration

Over the long term, it has been observed that regular exercise has a positive effect on the immune system in populations with an increased risk of CVD. Regular exercise can also help to reduce inflammation in the body and benefit the immune system. However, it is important to note that the specific effects of exercise on the immune system can vary depending on a variety of factors. Individuals who are already physically fit may not experience the same immune cell count boost as individuals who are less fit, as their immune systems may already be operating at a higher level or the body is already in a state of systemic low-grade inflammation. Next, the blood sampling time is crucial to ensure the blood samples are not taken at the wrong time, such as before the immune response has fully kicked in or after the peak response has already occurred. There may be other factors, such as pre-existing medical conditions or medications, that can affect the body’s immune response to exercise and lead to no changes or diagnostically lengthy alteration in immune cell count. It is important to note that a lack of change in immune parameters after exercise does not necessarily indicate a problem with the immune system, and there may be changes in other markers like insulin, cholesterol and glucose levels that are affected by exercise.

## Conclusion

The findings from the current studies suggest that exercises positively improve immune response outcomes. More importantly, this impact may improve the innate and adaptive immune response function as a potential anti-immunosenescent response to exercise in populations with increased CVD risk factors. Regardless of the types, intensity, and mode of exercise, it was well tolerated in populations with increased CVD risk factors and may have important implications for generating anti-inflammatory effects. The findings also indicate that upcoming studies must shift towards investigating exercise immunology at molecular levels to further justify its mechanism of action instead of simply measuring the circulating concentration.

## Figures and Tables

**Table 1:** Immune systems components

Innate components	Adaptive components
Cellular	Cellular
NK cells (CD16^+^, CF56^+^)	T-cells (CD3^+^, CD4^+^, CD8^+^)
Phagocytes (neutrophils, eosinophils, basophils, monocytes, macrophages)	B-cells (CD19^+^, CD20^+^, CD22^+^)

Soluble	Soluble
Acute-phase proteins	Immunoglobulins: IgA, IgD, IgE, IgG,
Complement	IgM
Lysozymes	
Cytokines (IL, IFN, CSF, TNF)	

Note: CD = clusters of differentiation; interleukin = IL; interferons = IFN; colony-stimulating factors = CSF; tumour necrosis factors = TNF; NK = natural killer

**Table 2:** Eligibility criteria of the identified studies according to PICO

Properties	Eligibility criteria
Type	Original research article
Language	English
Population	Adults (19 years old–59 years old) with increased risk of CVD risk factors; at least one of anthropometric indices (overweight/ obese BMI ≥ 25 kg/m^2^ or waist-to-hip ratio > 0.8 for females or > 0.9 for male) blood pressure ≥ 140/90 mmHg or hypertension fasting blood glucose ≥ 5.5 mmol or diabetes type II poor lipid profile (TC ≥ 5.2 mmol/L, HDL-C < 1.02 mmol/L, LDL-C ≥ 3.42 mmol/L and TG ≥ 1.72 mmol/L) sedentary/physically inactive/physical activity level < 150 min/week
Intervention	Exercise intervention such as aerobic, anaerobic, resistance, or combination etc.
Control/Comparator	Control group
Outcomes	Immune parameters such as WBC, monocyte, lymphocytes, T cells, neutrophils, NK cell, granulocytes, macrophages, IL-6, IL-10, IL-12, TNFα, NF-κB, TLR4, IFN-γ, s-IgA, IgA, IgG, IgM, thymus and spleen index

Note: BMI = body mass index; CVD = cardiovascular disease; TC = total cholesterol; HDL-C = high-density lipoprotein cholesterol; LDL-C = low-density lipoprotein cholesterol; TG = triglyceride; IL = interleukin; IFN = interferons; CSF = colony-stimulating factors; TNF = tumour necrosis factors

**Table 3:** Summary of outcome measures of included studies

Intervention outcome measure	Number of studies
White blood cell (WBC)/Leukocytes	111111
Monocytes/CD14^+^	111111111
Lymphocytes	111111
Neutrophils	1111
Thymus index	1
Spleen index	1
IL-6	111111
IL-10	1111
TNF-α	1111111
IFN-γ	1
T cell	11
NK cell	11
IgA	11
IgM	11

**Table 4:** The quality appraisal scores of each study

Study	C1	C2	C3	C4	Score
Peres et al. (15)	1	1	1	1	4
Blanks et al. (25)	1	1	1	1	4
Dorneles et al. (13)	1	0	1	1	3
Li and Ding (27)	1	1	0	1	3
Haley et al. (22)	1	1	1	1	4
Soltani et al. (20)	0	1	1	0	2
Barry et al. (23)	1	1	1	1	4
Mazur et al. (28)	0	1	1	1	3
de Souza et al. (11)	1	0	1	1	3
Yoon et al. (21)	1	1	1	1	4
Douglas et al. (26)	1	0	1	0	2
Gustafson et al. (14)	0	0	1	1	2
Rahim et al. (18)	1	1	1	1	4
Bartlett et al. (24)	1	0	1	1	3
Dorneles et al. (12)	1	0	1	1	3
Park et al. (17)	1	0	1	1	3
Silva-Reis et al. (19)	1	1	1	1	4
Rohnejad and Monazzami (16)	1	0	1	1	3

**Table 5:** Effects of exercise on immune parameters

Author, year	Study design	Sample size (male-female)	Study population (mean age)	Types of exercise	Comparable group	Intervention duration/time of blood sampling	Major findings
Peres et al., 2021	Non-randomised control trial	*N* = 40 (male)	Young sedentary males (25.8 ± 3.9 years old)	Aerobic: CPX on an electric treadmill ramp protocol	Sedentary lean group (*n* = 10), regular exercisers lean group (*n* = 10), sedentary obese group (*n* = 10), regular exercisers obese group (*n* = 10)	Acute: before, immediately, and 1 h after exercise	↑ WBC counts immediately after exercise and 1 h after exercise in all groups. ↑Monocyte counts immediately after exercise in all groups. ↑ Lymphocyte counts immediately after exercise and 1 h after exercise in all groups.
Li and Ding, 2020	Randomised control trial	*N* = 80 (44 males, 36 females)	Obese individuals (47.99 ± 7.49 years old)	Medium-intensity aerobic exercise (jogging, brisk walking, swimming and dancing)	Exercise intervention group (*n* = 40), basic intervention group (medication treatment) (*n* = 40)	Not mentioned	↑ Thymus index in both groups after intervention. ↑ Spleen index in both groups after intervention.
Haley et al., 2020	Randomised control trial	*N* = 116 (female)	Overweight premenopausal women at high risk for breast cancer	Home-based aerobic exercise on treadmill	Control group (*n* = 41), low-dose exercise group (*n* = 38), high-dose exercise group (*n* = 37)	Five menstrual cycles	↓TNFα in the high and low dose group. ↑ TNFα in the high-dose group. ↔ IL-10 in all groups.
Mazur et al., 2018	Randomised crossover	*N* = 31 (8 females, 23 males)	Adults with pre-hypertension (44.3 ± 5.57 years old)	Moderate-intensity aerobic training – cycling	Moderate intensity aerobic training group (*n* = 31), control group (*n* = 31)	12 weeks	↔ T cells subsets in all groups. ↔ Monocytes subsets in all groups. Limitation reported – heterogeneous group
Rahim et al., 2017	Randomised control trial	*N* = 44 (female)	Healthy sedentary females (29.7 ± 5.3 years old)	Aerobic dance exercise	Control group (*n* = 11), aerobic dance group (*n* = 11), combined aerobic dance exercise and honey supplementation group (*n* = 11), honey supplementation group (*n* = 11)	8 weeks	↓ WBC in D group. ↓ Neutrophils in D group. ↔ Lymphocytes in all groups. Limitation reported – small sample size.
Blanks et al., 2020	Non-randomised control trial	*N* = 24 (12 females, 12 males)	Healthy inactive individuals	Moderate-intensity cycle ergometer	HIACT group (*n* = 12), LOACT group (*n* = 12)	Acute: pre-, immediately post-, 1 h post- and 2 h post-exercise	↓ Monocytes CCR2 immediately post-exercise in HIACT.
Dorneles et al., 2020	Randomised crossover trial	*N* = 8 (male)	Obese males (28.42 ± 4.19 years old)	HIIE (running) and stepping up and down (exhaustive exercise)	HIIE (*n* = 8), exhaustive exercise group (ES) (*n* = 8)	Acute: before and immediately after exercise	↑ IL-6 after exercise in both groups. ↑ IL-10 after exercise only in HIIE. ↑TNF-α after exercise in ES.
Rohnejad and Monazzami, 2023	Randomised control trial	*N* = 22 (male)	Overweight middle-aged men	HIIT running on a treadmill	Training (*n* = 12), control (*n* = 10) groups	Acute: pre-, 1 h, 24 h and 48 h post-exercise	↑ IL-6 serum levels in one-hour and 24 h phases after the HIIT training compared to pre-test.
Barry et al., 2018	Randomised control trial	*N* = 33 (5 males, 28 females)	Inactive adults with obesity	HIIT, MICT – cycling	HIIT group (*n* = 16), MICT group (*n* = 17)	2 weeks	↔ IL-10 in both groups. ↔ IL-6 in both groups. ↔ TNFα in both groups. ↔ T cells in both groups. ↔ Neutrophils in both groups. ↔ CD14^+^ monocytes in both groups. ↔ WBC in both groups. Limitations reported – subtle changes within participants’ diet may influence the result, unequal representation of males and females, unstandardised female menstrual phase (menopausal, menses) during pre- and post-tests.
de Souza et al., 2018	Randomised crossover trial	*N* = 10 (male)	Sedentary obese males (28.5 ± 2.7 years old)	Low-volume HIIE, MICE – running	Low-volume HIIE (*n* = 10), MICE (*n* = 10), Rest-control (*n* = 10)	Acute: before, immediately after exercise and 1 h post-exercise	↓ IFN-γ immediately after HIIE and remained lower 1 h post-HIIE. ↑ IFN-γ immediately after MICE and 1 h post MICE. ↑ IL-6 immediately after both HIIE and MICE and 1 h post MICE and HIIE.
Douglas et al., 2017	Non-randomised control trial	*N* = 20 (8 males, 12 females)	Overweight to obese adults: Active (27 ± 1 year old) versus sedentary (24 ± 2 years old)	Aerobic exercise – moderate to vigorous intensity – treadmill or cycle ergometer	Active (*n* = 8), sedentary (*n* = 12)	Acute: pre- and 1 h post-exercise	↔ TNF-α in both groups. ↔ CD14^+^ in all groups. ↓ IL-6 in active groups. Limitations reported – small number of subjects, different volume and intensity of exercise training.
Barlett et al., 2017	Randomised control trial	*N* = 20 (8 males, 12 females) N = 27 (9 males, 18 males)	Healthy inactive individuals (43 ± 11 years old)	HIIT, MICT – cycling	HIIT (*n* = 14), MICT (*n* = 13)	10 weeks	↑ Monocyte phagocytosis in both groups. ↓ CD14^+^/CD16^+^ monocytes % in HIIT. ↔ Total lymphocytes in both groups. ↔ Total WBC in both groups. ↔ Neutrophils in both groups. ↔ IL-6 in both groups. ↔ TNFα in both groups.
Dorneles et al., 2016	Non-randomised control trial	*N* = 22 (male)	Lean and overweight obese males (27.41 ± 9.20 years old)	MIIE, HIIE - cycling	Lean (*n* = 10), overweigh-obese (*n* = 12)	Acute: pre-, immediately and 30 min post-exercise	↑ WBC immediately post-exercise in both groups. ↔ WBC 30 min post-exercise in both groups. ↑ Lymphocytes immediately post-exercise in the overweight-obese group. ↑ Monocytes immediately post-exercise in both groups.
Soltani et al., 2020	Cross-sectional nonrandomised controlled	*N* = 30 (female)	Obese young females	CHIIT – aerobic and resistance training	Control group (*n* = 15), Exercise group (*n* = 15)	10 weeks	↓ TNFα level in EG. ↓ TLR4 level in EG.
Yoon et al., 2018	Randomised control trial	*N* = 30 (female)	Obese post-menopausal women	Aerobic exercise (walking on the treadmill), Resistance exercise (bench press, lat-pull down, triceps, push-down, dumbbell curl, sit-up, squat, leg extension, leg flexion, and leg press)	Aerobic exercise group (*n* = 10), resistance Exercise group (*n* = 10), Control group (*n* = 10)	12 weeks	↑ NK cell activity in all groups. ↔ IgA in all groups. ↓ IgG in aerobic and resistance groups. ↑ IgG in the control group. ↓ IgM in aerobic and resistance groups. Limitations reported – did not include the amount of physical activity, smoking, drinking, dietary habits and stress level into consideration.
Park et al., 2015	Randomised control trial	*N* = 20 (female)	Post-menopausal middle-aged women with abdominal obesity	Combined aerobic exercise and resistance training	Exercise group (*n* = 10), Control group (*n* = 10)	12 weeks	↓ TNFα in the exercise group. ↑ IgA in the exercise group. ↓ monocytes in the exercise group. ↔ IgM in all groups.
Silva-Reis et al., 2022	Non-randomised control trial	*N* = 41 (female)	Overweight and obese women	Combined physical exercise (aerobic + resistance training)	Non-obese (*n* = 12), overweight (*n* = 17), obese grade I (*n* = 11)	12 weeks	↑ IL-10 in non-obese and obesity grade I groups ↓leukocytes in non-obese and obesity grade I groups ↓neutrophils in non-obese and obesity grade I ↑lymphocytes in non-obese and obesity grade I ↓monocytes in overweight and obesity grade I
Gustafson et al., 2017	Non-randomised crossover experimental study	*N* = 15 (male)	Male population of varying fitness	Visit 1 - a brief maximal intensity cycling regimen; Visit 2 - less intense endurance cycling regimen	Maximal intensity cycling (*n* = 15) versus Less intense endurance cycling (*n* = 15); active (*n* = 10) versus sedentary (*n* = 5)	Acute: before exercise, 2 min– 5 min post-exercise, 3 h post-exercise and 24 h post-exercise	↑ Lymphocytes 2 min–5 min post in maximal group. ↑ Monocytes 2 min–5 min post in maximal group. ↑ NK Cell 2 min–5 min post in maximal group.

Notes: CPX = cardiopulmonary exercise testing; HIIE = high intensity interval exercise; HIIT = high intensity interval training; CHIIT = combined all-extremity high-intensity interval training; MICT = moderate intensity continuous training; MICE = moderate intensity continuous exercise session; MIIE = moderate intensity interval exercise; HIACT = high physically active; LOACT = low physically inactive; WBC = white blood cell; NK = natural killer; IL-6 = interleukin 6; IL-10 = interleukin 10; ; IL-12 = interleukin 12; TNFα = tumour necrosis factor-alpha; NF-κB = nuclear factor Kappa-light-chain-enhancer of activated B cells; IFN-γ = interferon Gamma; TLR4 = toll like receptor 4 ; T cells = T lymphocyte; s-IgA = secretory immunoglobulin A; IgG = immunoglobulin G; IgM = immunoglobulin ; ↑ = significant increase; ↓ = significant decrease; ↔ = no change

**Table 6:** Immune actions of selected parameters

Immune parameters	Immune actions
Leukocytes	
Neutrophils	Phagocytosis
Monocytes	Phagocytosis; develops into macrophages in tissue
Cytokine	
IL-6, IL-10	Stimulates the differentiation of B-cells; inflammation and acute-phase response
TNF-α, IFN-γ	Enhances tumour cell killing and antiviral activity
Lymphocytes subset	
T cells (CD3^+^)	Recognise antigen to coordinate acquired response
Th cells (CD3^+^ CD4^+^)	Secrete cytokines that stimulate T- and B-cell proliferation and differentiation
B cells (CD19^+^, CD20^+^, CD22^+^)	Produce and secret Ig specific to activate antigen; exhibit memory
NK cells (CD3−,CD16^+^, CD56^+^)	Trigger by IgG; control foreign materials until the antigen-specific immune system responds
Immunoglobulin	
IgM	Early immune response; stimulation of ingestion by macrophages
IgG	Stimulation of ingestion by macrophages
IgA	Localised protection in external secretion, e.g. saliva
